# A paternal effect of MTHFR SNPs on gametes and embryos should not be overlooked: case reports

**DOI:** 10.1007/s10815-019-01488-9

**Published:** 2019-05-22

**Authors:** Laetitia Jacquesson-Fournols, Silvia Alvarez, Marc Cohen, Patrice Clement, Yves Menezo

**Affiliations:** 1 Paris, France; 2 Gyn Obst Clinique de La Muette, 75116 Paris, France; 3 Gyn Obst, Clinique Natecia, Lyon, France; 4 Laboratoire Clement, 17 Avenue d ‘Eylau, 75016 Paris, France

## Introduction

**Fig. 1:**
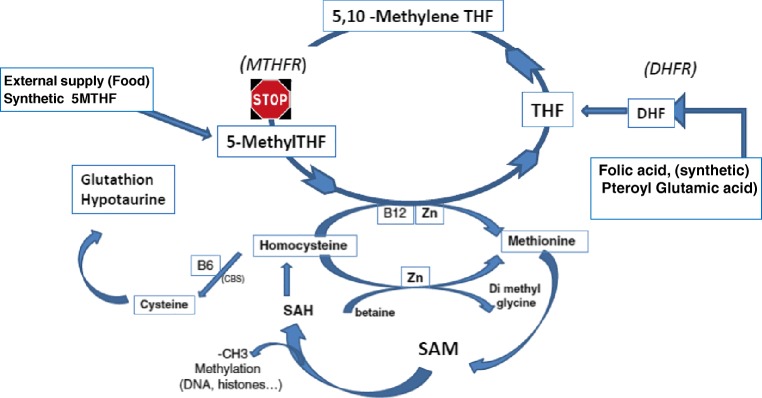
The folates and the one-carbon cycle (1-CC). The synthetic folic acid enters the folates cycles after a 2-step modification by DHFR: dihydrofolate reductase. In MTHFR SNPs carriers, the capacity to form 5 MTHF drops down to 75% in 677TTs. 5MTHF is located downstream the MTHFR enzyme. Then, Hcy recycling is severely impaired. Note that the CBS pathway is allosterically activated by SAM (SAdenosyl Methionine): this pathway is of major importance for the synthesis of glutathione and Hypotaurine two regulators of oxidative stress

The process of methylation is a major biochemical component of all cells, essential in allowing covalent linking of methyl groups to lipids, proteins, and nucleic acids. It is involved in the regulation of membrane transport and in neurotransmitter function. The interaction between DNA and methylation is of fundamental importance as a major factor in imprinting and epigenesis, regulating gene expression without modification of the underlying DNA sequence. In reproductive physiology, de-methylation/re-methylation processes are very active and dynamic, especially during gametogenesis and embryogenesis. Methylation is initially maintained during early preimplantation stages, depending of the oocyte endogenous pool and the maternal capacity to supply methyl donors during the preimplantation stages of development [1]. The majority of methyl tags are later erased then restored in germinal cells, during prenatal life in males and during post-natal follicle development in females. Oxidative stress, whether or not related to the environment, has an effect on these regulatory methylation processes [2, 3] and is a major contributor to an observed decrease in fertility [4]. As early as the preimplantation stage, the embryos must guard against potential insults affecting methylation. MethyleneTetraHydroFolate Reductase (MTHFR) SNPs (Single Nucleotide Polymorphism see Fig. 1), especially the C677T, is a significant inducer of perturbations in methylation that affect fertility. This is now well established for women [5, 6] as the preimplantation development is, during the 3–4 days totally dependent of the oocyte quality in term of maternally derived mRNAs and metabolites and nutrients supplied by  the oviduct. 677TT homozygous women have a significant decrease in early preimplantation embryo developmental capacity, as measured by PGS (preimplantation genetic screening) [5]. In men, a strong trend in the relationship between MTHFR SNPs and fertility has been demonstrated [7]. In this report, we describe the negative effect of a paternal C677CT MTHFR SNP. We will also confirm that synthetic folic acid is not a therapeutic option for male MTHFR carriers, confirming the observations of El Aarabi et al. [8] with respect to both men and women: Folic acid (FA: Pteroyl Glutamic acid) fortification should not be considered as a dogma, but should instead be considered within the perspective of its interaction with the underlying subject genetics.

## Cases description

1. *Couple MT/LF*: The female partner was born in February 1978 and her husband in January 1977. The couple underwent 6 cycles of IVF, with one biochemical pregnancy and an ectopic pregnancy, treated with Methotrexate. During all of the IVF attempts, she was primed with either low (0.4 mG/day) or high (5 mG/day) doses of Folic acid. MTHFR testing indicated that she was heterozygous for C677T SNP, and she started a series of oocyte donation (OD) cycles. The first cycle in 2015 was successful, with the birth of a male baby. She then continued OD cycles unsuccessfully, with one biochemical pregnancy early in 2017. She was then referred to our group, after another OD failure primed with high doses of Folic acid (5 mG/day). When she came to us, in view of being heterozygous for MTHFR C677T, she was started on 5 Methyl Tetrahydrofolate, 400 μG daily (the folate compound downstream of MTHFR), sustained by a support of the one-carbon cycle (Zn, B3, B6, B12, Impryl®, Parthenogen, Lugano, Switzerland). In our practice, this supplement is systematically administered to all patients who carry a MTHFR SNP. She then undertook a cycle with transfer of blastocyst, frozen during the previous (3rd) OD cycle, treated with Impryl® and Heparin (Lovenox®): this cycle ended with a miscarriage at 8.5 weeks. The couple then decided on a further OD cycle, with the female partner continuing Impryl® pre-treatment for 3 months. No one of the oocyte donors (from Spain and US) had been tested for these MTHFR SNPs. The women continued the treatment after the last OD failures. Her partner has normal sperm parameters according to WHO criteria, and he was tested for MTHFR SNPs C677T and A1298C. He was found to be homozygous for MTHFR 677TT (with no A1298 SNP variant), and was immediately started on 5-MTHF folate treatment at the same dose as his wife. The couple conceived spontaneously 8 weeks later. The treatment was continued throughout pregnancy and during breast feeding. A female baby was delivered at 38 weeks in December 2018, weighing 2.7 kg, 49 cm in length.
2. *Second case series*: When couples have high difficulties to conceive, whatever the woman genetic profile, the man is systematically tested. In seven couples for whom the woman had been tested WT or HTZ for one mutation, treatment did not allow them to conceive. When tested, the men had three mutated alleles (two 677TT/1298AC and five 1298CC/677CT), with no elevation of the circulating homocysteine. For another couple, the woman was tested 1298CC/677CT and her companion has been tested 677TT/1298AC with an elevated homocysteine (Hcy: 20μMoles per liter). Treatments had been unsuccessful for all these couples. A decrease in Hcy from 20 to 11 μmol per liter was observed for the man who had an elevated Hcy

## Conclusion

Our observations confirm the concept that a correct process of methylation is mandatory for complete gametogenesis and embryonic development. Although the maternal impact of MTHFR SNPs is important and has been clearly demonstrated at early and late preimplantation stages [5, 6], a paternal effect should not be overlooked. The paternal effect is most likely delayed in terms of a negative impact on embryo development, probably by affecting trophoblast function. The second aspect for consideration is that high doses of folic acid do not allow correct embryonic development if the gametes are “methylation defective” with respect to MTHFR mutations. The oocyte is apparently unable to correct methylation errors carried by sperm. Testicular methylation deficiency is not corrected by exogenous folic acid support which rather may exacerbate the problems [8, 9] It seems that folic acid is not helpful in ovarian and testis methylation deficiencies linked to MTHFR SNPs. 5-MTHF, the metabolite downstream of MTHFR, can apparently avoid these methylation errors in MTHFR SNP carriers. It also helps in regulating sperm methylation. This has been observed for 677TT male, whatever the genetic background of the woman. Four deliveries have been obtained in a series (already partly published [10]), of 6 couples where both members were 677TT. However, when the men are carriers of three mutated alleles**,** the situation looks so far more complicated: these patients look resistant to the treatments. This type of genetic background has not been described in the UK study [5], but is rather frequent (it can reach 5% of the population**)** in the south of Italy [11].

The multiple oocyte donation failures observed in the first case raise three issues: 1/Oocyte donation in not necessarily an inevitable path after multiple IVF failures. 2/Oocyte donors should be tested for these SNPs, as the success in one of four oocyte donations may be related to the fact that the oocyte donor was completely free of these SNP variants. 3/The male partner should be tested after ART failures, especially if the woman has tested positive. One must not forget that the live birth rate per oocyte retrieved in donors is below 10% [12] and that the prevalence of MTHFR SNPs in the Caucasian and Han populations is over 50%. When tested positive, patient treatment with 5MTHF (Impryl® or Tetrafolic®) should increase the chance of having a baby and also avoid also the expensive, time**-**consuming**,** and sometimes deleterious process of PGS.

## References

[1] Ménézo Y, Clément P, Dale B (2019). 2019 DNA methylation patterns in the early human embryo and the epigenetic/imprinting problems: a plea for a more careful approach to human assisted reproductive technology (ART). Int J Mol Sci.

[2] Tunc O, Tremellen K (2009). Oxidative DNA damage impairs global sperm DNA methylation in infertile men. J Assist Reprod Genet.

[3] Menezo YJ, Silvestris E, Dale B, Elder K (2016). Oxidative stress and alterations in DNA methylation: two sides of the same coin in reproduction. Reprod BioMed Online.

[4] Manikkam M, Tracey R, Guerrero-Bosagna C, Skinner MK (2013). Plastics derived endocrine disruptors (BPA, DEHP and DBP) induce epigenetic transgenerationnal inheritance of obesity, reproductive disease and sperm epimutations. PLoS One.

[5] Enciso M, Sarasa J, Xanthopoulou L, Bristow S, Bowles M, Fragouli E, Delhanty J, Wells D (2016). Polymorphisms in the MTHFR gene influence embryo viability and the incidence of aneuploidy. Hum Genet.

[6] Goyco Ortiz LE, Servy EJ, Menezo YJR (2019). A successful treatment with 5 methyltetrahydrofolate of a 677 TT MTHFR woman suffering premature ovarian insufficiency post a NHL (non-Hodgkin's lymphoma) and RPL (repeat pregnancy losses). J Assist Reprod Genet.

[7] Gong M, Dong W, He T, Shi Z, Huang G, Ren R, Huang S, Qiu S, Yuan R (2015). MTHFR 677C>T polymorphism increases the male infertility risk: a meta-analysis involving 26 studies. PLoS One.

[8] El Aarabi M, San Gabriel MC, Chan D, Behan NA, Caron M, Pastinen T, Bourque G, MacFarlane AJ, Zini A, Trasler J (2015). High-dose folic acid supplementation alters the human sperm methylome and is influenced by the MTHFR C677T polymorphism. Hum Mol Genet.

[9] El Aarabi M, Christensen KE, Chan D, Leclerc D, Landry M, Ly L, Rozen R, Trasler J (2018). Testicular MTHFR deficiency may explain sperm DNA hypomethylation associated with high dose folic acid supplementation. Hum Mol Genet.

[10] Servy EJ, Jacquesson-Fournols L, Cohen M, Menezo YJR (2018). MTHFR isoform carriers. 5-MTHF (5-methyl tetrahydrofolate) vs folic acid: a key to pregnancy outcome: a case series. J Assist Reprod Genet.

[11] Zappacosta B, Graziano M, Persichilli S, Di Castelnuovo A, Mastroiacovo P, Iacoviello L (2014). 5,10-Methylenetetrahydrofolate reductase (MTHFR) C677T and A1298C polymorphisms: genotype frequency and association with homocysteine and folate levels in middle-southern Italian adults. Cell Biochem Funct.

[12] Martin JR, Bromer JG, Sakkas D, Patrizio P (2010). Live babies born per oocyte retrieved in a subpopulation of oocyte donors with repetitive reproductive success. Fertil Steril.

